# Cost-effectiveness of *Lifestyle Africa*: an adaptation of the diabetes prevention programme for delivery by community health workers in urban South Africa

**DOI:** 10.1080/16549716.2023.2212952

**Published:** 2023-05-23

**Authors:** Melanie D. Whittington, Kathy Goggin, Lungiswa Tsolekile, Thandi Puoane, Andrew T. Fox, Ken Resnicow, Kandace K. Fleming, Joshua M. Smyth, Frank T. Materia, Emily A. Hurley, Mara Z. Vitolins, Estelle V. Lambert, Naomi S. Levitt, Delwyn Catley

**Affiliations:** a Department of Population Health, University of Kansas Medical Center, Kansas City, KS, USA; b Department of Pediatrics, University of Missouri – Kansas City School of Medicine, Kansas City, MO, USA; c Health Services and Outcomes Research, Children’s Mercy Hospitals and Clinics, Kansas City, MO, USA; d School of Public Health, University of the Western Cape, Cape Town, South Africa; e School of Public Health, University of Michigan, Ann Arbor, MI, USA; f Life Span Institute, University of Kansas, Lawrence, KS, USA; g College of Health and Human Development, Penn State University, University Park, PA, USA; h Department of Epidemiology & Prevention, Wake Forest School of Medicine, Winston-Salem, NC, USA; i UCT Research Centre for Health through Physical Activity, Lifestyle and Sport (HPALS), Division of Research Unit for Exercise Science and Sports Medicine, Faculty of Health Sciences, University of Cape Town, Cape Town, South Africa; j Department of Medicine and Chronic Disease Initiative for Africa, Faculty of Health Sciences, University of Cape Town, Cape Town, South Africa; k Center for Children’s Healthy Lifestyles and Nutrition, Children’s Mercy Kansas City, Kansas City, MO, USA

**Keywords:** Economic analysis, HbA1c, lifestyle interventions, low- and middle-income countries, probabilistic sensitivity analysis

## Abstract

**Background:**

*Lifestyle Africa* is an adapted version of the Diabetes Prevention Program designed for delivery by community health workers to socioeconomically disadvantaged populations in low- and middle-income countries (LMICs). Results from the *Lifestyle Africa* trial conducted in an under-resourced community in South Africa indicated that the programme had a significant effect on reducing haemoglobin A1c (HbA1c).

**Objective:**

To estimate the cost of implementation and the cost-effectiveness (in cost per point reduction in HbA1c) of the *Lifestyle Africa* programme to inform decision-makers of the resources required and the value of this intervention.

**Methods:**

Interviews were held with project administrators to identify the activities and resources required to implement the intervention. A direct-measure micro-costing approach was used to determine the number of units and unit cost for each resource. The incremental cost per one point improvement in HbA1c was calculated.

**Results:**

The intervention equated to 71 United States dollars (USD) in implementation costs per participant and a 0.26 improvement in HbA1c per participant.

**Conclusions:**

*Lifestyle Africa* reduced HbA1c for relatively little cost and holds promise for addressing chronic disease in LMIC. Decision-makers should consider the comparative clinical effectiveness and cost-effectiveness of this intervention when making resource allocation decisions.

**Trial Registration:**

Trial registration is at ClinicalTrials.gov (NCT03342274).

## Introduction

The combination of poor diet and sedentary lifestyle places an ever-increasing number of people at risk for developing type 2 diabetes (T2DM) and/or cardiovascular disease (CVD) [1,2]. The global economic burden of T2DM and CVD is expected to be in the trillions of US dollars by 2030 [3,4]. Approximately 75% of those with T2DM live in low- and middle-income countries (LMICs) [5]. In South Africa, addressing CVD and T2DM has been identified as an urgent priority by public health authorities given rates of high blood pressure and rising obesity trends [6,7]. Prevention of metabolic syndrome via lifestyle intervention for those at risk is possible, as shown by the studies of the Diabetes Prevention Program (DPP) [8]. Because of both the high intensity of the DPP and the fact that underserved populations tend to be at greater risk for T2DM/CVD, the DPP has frequently been adapted from its original form to increase the ease of dissemination (e.g. reduced need for involvement of primary care, lower cost) and cultural sensitivity. Cost evaluations of DPP adaptations have also been conducted in high-income countries, but much less is known about costs in LMICs, where the economic burden of metabolic syndrome is increasing even more rapidly. Two lifestyle intervention studies in LMICs utilising peer leaders in India and trained community members in South Africa found the programmes to likely be cost-effective with costs of about $20 per participant [9,10].

We recently developed and evaluated *Lifestyle Africa* a culturally tailored, video-based, community health worker (CHW)-delivered adaptation of the DPP [11]. It was specifically designed to be suitable for socio-economically disadvantaged communities and to be scalable in LMIC contexts. The 17-session programme was evaluated in a cluster-randomised clinical trial that compared *Lifestyle Africa* to usual care in a low-resource, predominantly Black community in Cape Town, South Africa [11]. Most intervention sessions were monitored for fidelity by study staff and found to be adequate. Attendance was acceptable particularly considering the barriers faced by residents in a low-resource setting. Participants’ rated acceptance of the programme was high.

This trial represents a significant test of the reach of the DPP by using CHWs in a low-resource environment in an LMIC with extensive cultural adaptation. It is noteworthy that this intervention was conducted outside of the primary care setting and delivered by trained laypeople (i.e. not study staff) and was nonetheless able to show some benefit in lowering HbA1c, one of the major factors contributing to metabolic syndrome [11]. T2DM/CVD morbidity is extremely costly, and cost-effective mitigation strategies will become increasingly important [12,13]. Governments and NGOs will be more likely to allocate needed funds for programmes with both proven benefit and low cost. The objective of this evaluation was to estimate the cost of implementing and the cost-effectiveness of the *Lifestyle Africa* programme to inform decision-makers of the resources required to implement and the value of this intervention.

## Methods

Procedures and methods of the *Lifestyle Africa* trial and details on intervention development have been reported previously in detail [14,15]. Here we provide a brief overview of the methods. The study was approved by the Institutional Review Boards at both the University of Cape Town (primary) and Children’s Mercy Kansas City and is registered with ClinicalTrials.gov (NCT03342274).

### Study design and participants

The study was a two-arm, parallel group, cluster-randomised controlled trial. Participants were enrolled in two waves separated by a year. Each wave completed baseline assessments in February and March of the enrolment year. Participants in both arms completed follow-up assessments at convenient locations approximately 8 months post-enrolment. Participants received R150 (approximately 9.60 USD) in the form of a gift voucher for completing each assessment.

The study took place in an under-resourced urban township outside of Cape Town, South Africa. Almost all residents are Xhosa-speaking Black South Africans facing high levels of poverty and unemployment, low education and rapidly rising rates of overweight and obesity [16,17]. The trial was conducted in partnership with two non-governmental organisations (NGOs). The NGO’s health services were delivered via community healthcare workers (CHWs) who were members of the community with limited basic medical training. CHWs provide services at local health ‘club’ locations which are commonly situated in churches, community buildings or homes and where daily to weekly group meetings were held.

After conducting video-based recruitment sessions, study staff and CHWs screened 782 individuals from the pre-existing community groups between February 2018 and March 2019. A total of 494 participants met the inclusion criterion of being overweight/obese (i.e. body mass index [BMI] ≥ 25 kg/m^2^). Exclusion criteria included uncontrolled high blood pressure; HbA1c > 11; being pregnant, breastfeeding or planning pregnancy; use of medicine that might affect weight loss; planning to leave the club before the end of the trial and possessing intellectual disabilities that might interfere with programme participation.

Clusters consisted of ‘CHW teams’ (as opposed to individual CHWs or clubs) because some CHWs worked in pairs or trios and sometimes with more than one club. The clusters were stratified by NGO and allocated using a 1:1 scheme where the club would either receive the *Lifestyle Africa* intervention in addition to usual care or receive usual care for 1 year until crossover in year 2. The study statistician randomised clusters using the list of CHWs teams and a computer-based number generator. The nature of the trial did not allow for blinding of CHWs, participants or research staff.

### Usual care and the Lifestyle Africa intervention description

CHWs’ usual care to clubs involved approximately monthly monitoring of weight, blood pressure and blood glucose, along with medication delivery and referring patients for additional care when needed. CHWs assigned to the intervention arm provided usual care as well as the *Lifestyle Africa* intervention. The *Lifestyle Africa* intervention was a culturally adapted version of the DPP designed to be appropriate for participants living in under-resourced Xhosa-speaking communities in South Africa. Preliminary formative research was conducted in collaboration with two community advisory boards to inform development of a curriculum and materials that would be acceptable to the target population. The programme consisted of 17 weekly in-person sessions facilitated by trained CHWs. The content of the video-based curriculum focused on central tenets from the DPP including increasing and tracking physical activity, nutrition, stress management and other lifestyle modifications associated with weight loss. Participants received workbooks, handouts and activities in either Xhosa or English. Participants were also given the opportunity to enrol in a custom-built text messaging (short message service; SMS) system that would push semi-tailored SMS to their personal mobile phones. Messages were delivered throughout the week between programme meetings and focused on programme reminders, motivation for achieving goals, self-efficacy boosters and affirmations.

### Outcome measures

The primary outcome was weight loss from baseline to the end of the year, and secondary outcomes were blood pressure, HbA1c, low-density lipoprotein and triglycerides. Because the main outcome analysis from the trial indicated that the only significant effect of the intervention was on HbA1c, this study only examines this outcome.

### Cost collection approach and analysis plan

The analytic approach followed best practices for conducting and reporting economic evaluations alongside behavioural health programmes, including recommendations from the Centers for Disease Control and Prevention and the Consolidated Health Economic Evaluation Reporting Standards (CHEERS) [18,19]. To estimate the implementation costs, study records (e.g. attendance records, club site locations) and key informant interviews with NGO partner administrative and management staff were conducted to identify the activities and resources required to implement the intervention. A direct-measure micro-costing approach was used to determine the number of units and unit cost for each resource. The perspective of the analysis was that of a non-government organisation, or some other organisation that would deliver this developed intervention. The time horizon was approximately 8 months, representing the length of follow-up of the trial. Costs to develop the intervention were intentionally not included because now that the programme has been developed, it is freely available to organisations who want to implement this intervention. Therefore, there are no costs associated with programme development for organisations wishing to implement this intervention. Programme development costs might be needed for adaptation of the programme to a new setting (e.g. language translation, local photography), but these costs would be marginal and dependent on how different the implementation setting is from the setting of the initial programme development. All costs were converted to 2020 USDs. Costs were divided into two categories: start-up costs and implementation costs. Start-up costs were defined as the one-time resources needed to initiate the intervention: the items that could be reused for multiple intervention implementations. Start-up costs included the CHW training and the purchase of the equipment necessary to deliver the sessions, including the one-time SMS system deployment fee. Implementation costs were defined as the resources necessary to deliver the components of the intervention: the items that would be required each time the intervention is implemented. Implementation costs included the personnel time to deliver and support the session delivery, as well as the SMS costs to send the messages to patients and the monthly maintenance fees. Start-up and implementation costs could include personnel and non-personnel resources.

The start-up costs were identified and reported per club (i.e. where sessions were delivered) due to most of the start-up costs being fixed costs unrelated to the number of participants. The implementation costs were reported per club and per participant given a mixture of fixed and variable (i.e. dependent on the number of participants) costs. The per club implementation costs were divided by the average number of participants to estimate the per participant cost. Data on the personnel resources were collected through prospective time logs of the session delivery collected on a subsample of sessions (mean of 3 for each of the 17 sessions) and through interviews with project personnel. The average time reported on the time logs was multiplied by the average hourly wage (inclusive of salary and benefits) for the respective occupation. Non-personnel resources were tracked and recorded through project receipts and invoices. The cost-effectiveness of the intervention was calculated by dividing the average per participant implementation cost by the average per participant improvement in HbA1c to calculate the incremental cost per one-point reduction in HbA1c. Start-up costs were outside of the costs included in the cost-effectiveness analysis because they represent fixed costs (i.e. do not vary based on the number of participants or the number of times it is implemented) and can be reused across multiple implementations. To identify the parameters with the most influence on the cost-effectiveness, a one-way sensitivity analysis was conducted that varied each model input among its upper and lower bound. To account for parameter uncertainty and variation, a probabilistic sensitivity analysis was conducted by assigning appropriate distributions (gamma for values greater than 0 and beta for values between 0 and 1) to each input and then running 1,000 iterations of the analysis. To estimate the lower and upper bounds used in the one-way sensitivity analysis and the distribution used in the probabilistic sensitivity analysis, we first assigned a standard error to each model parameter. The standard errors for the HbA1c values were captured as part of the trial data collection efforts and were approximately 0.06 for the intervention and 0.05 for the comparator. For the cost inputs (e.g. unit costs, hourly wages), we assumed a standard error that was 10% of that of the average unit cost. For example, the average unit cost for a participant handbook was $13.25, and thus we assumed a standard error of 1.32, which resulted in a credible range for the participant handbook cost parameter of $10.78 to $15.97. The average unit cost was used in the base-case analyses, whereas the distribution around the parameter was used in the sensitivity analyses.

## Results

### Key results from the prior main analysis of the trial

Results previously reported [11] indicated that the intervention was feasible (e.g. modal number of sessions held by CHWs was 17, 42% of participants attended at least 75% of sessions held) and mean completion rates of required facilitation elements were 85.4%. The average duration of the intervention was 6.4 months; this includes holidays, cancellations and rescheduling for inclement weather, and other disruptions. The participants in the Lifestyle Africa trial were generally low-socio-economic status, older women (mean age = 67.71, 88.7% female, 47.5% with lower than Grade 8 education). The participants were all overweight or obese (mean weight = 85.28 kg, BMI = 34.54) and 22% were taking diabetes medication at baseline, and mean HbA1c was 6.32. Baseline characteristics of participants were similar between groups. Although the intervention did not have a significant effect on weight loss, ANCOVA models that adjusted baseline values and clustering indicated that it did result in a statistically significant reduction in HbA1c among intervention participants relative to control participants (6.23 [95% CI = 6.12, 6.34] for intervention versus 6.49 [95% CI = 6.37, 6.58] for controls; mean difference = 0.26). The effect was approximately one-quarter of the effect observed for metformin in randomised trials [20].

### Cost of implementation

Following key informant interviews with NGO partner administrative and management staff, a detailed description of the resources necessary to initiate and implement the intervention was created. Table 1 details the number of units and unit cost for each non-personnel and personnel resource required. The number of units are reported per club (i.e. group/site that delivered the 17 sessions of content). In our study, there was an average of 17 clubs per non-government organisation, an average of 14 participants per club and an average of 1.5 CHWs per club who led the intervention sessions. The values provided in Table 1 were used to calculate the incremental implementation costs for *Lifestyle Africa*.

**Table 1.** Resources to implement *Lifestyle Africa*, per club.

Non-personnel resources	Number of units per club	Average* unit cost (USD^†^)	Notes
Training materials	1.5	$11.78	1 package of training materials for each community health worker
Scale	1	$38.40	1 scale for every 15 participants
VIVITEK Qumi 6 mini projector	1	$717.60	
VIVITEK Qumi battery pack	1	$163.02	
Small speaker	1	$38.98	
Large portable speaker	1	$120.34	
Box of pens	1	$9.54	
Measuring cups and spoons	1	$7.04	
Flip chart stand	1	$91.52	
Flip chart paper	1	$9.09	
Participant handbook	14	$13.25	Number of units is equivalent to the average number of participants per club.
Stickers	14	$0.06	
Certificates	14	$0.44	
Weight log	14	$0.22	
SMS system	0.06	$13,200.32^††^	1 SMS is needed per organisation; thus, the number of units is equivalent to 1 divided by the average number of clubs per organisation ( = 1/17). Unit cost is the one-time deployment cost.
SMS messages	493 per participant	$0.01	On average, there were 14 participants per club.
SMS short code fee	Monthly	$2.15	For 6.4 months based on the average duration of the intervention.
Agile system maintenance	Monthly	$59.75	
Personnel resources	Personnel per club	Hourly wage (USD*)	Notes
Community health workers	1.5	$2.01	68 h for training; 85.5 hfor session delivery (4.5 h per session for 19 sessions).
Trainer	0.1	$0.90	1 trainer per 15 community health workers; 68 hours for training.
Manager	2 h per month	$6.79	Supervised overall operation of the program at NGO.
Nurse coordinator	2 h per month	$4.74	Provided oversight of CHWs work with clubs.
Administrative coordinator	6 h per month	$2.80	Coordinated schedules, collected attendance records and prepared session materials and equipment for CHWs.

*This table reports the average unit costs modeled in the base-case analysis. In sensitivity analyses, these average costs were varied assuming a standard error that was 10% of the average unit value.

^†^In 2020 the conversion from South African Rands (R) to United States dollars (USD) was 0.064. Thus, R figures were multiplied by 0.064 to estimate USD. Technology costs are high due to high South African import duties on these items.

^††^This unit cost represents the unit cost for the SMS system selected for programme implementation within this trial, which is a US commercial product with rich automated scheduling and other features. There are much less expensive options that can deliver messages on a schedule such as WhatsApp with a third-party WhatsApp scheduler application that costs less than $5.00.

Table 2 details the average intervention costs stratified by start-up and implementation costs. Start-up costs equated to nearly $2,400, with most of these costs used to purchase the necessary equipment to deliver the intervention. Implementation costs were approximately $988 per club for the 17-week intervention to be implemented. This equated to an average per participant implementation cost of $71. Nearly half of these costs were to support the personnel necessary for the implementation of the programme and the other half of these costs were to provide the text messages to the participants through the SMS system.

**Table 2.** Average per club intervention costs (USD).

Activity	Personnel costs per club (95% CI)	Non-personnel costs per club (95% CI)	Total cost per club (95% CI)	Total cost per participant (95% CI)
Start-up				
Training	$211 ($137, $310)	$18 ($15, $21)	$229 ($156, $325)	N/A
Equipment	N/A	$1391 ($1,238, $1,560)	$1391 ($1,238, $1,560)	N/A
SMS	N/A	$776 ($634, $928)	$776 ($634, $928)	N/A
*Subtotal*	**$211** ($137, $310)	**$2185** ($1,973, $2,416)	**$2396** ($2,171, $2,643)	**N/A**
Implementation				
SMS	N/A	$476 ($357, $610)	$476 ($357, $610)	$34 ($25, $44)
Sessions	$258 ($158, $405)	N/A	$258 ($158, $405)	$18 ($11, $29)
Additional Personnel	$255 ($197, $331)	N/A	$255 ($197, $331)	$18 ($14, $24)
*Subtotal*	**$513** ($395, $690)	**$476** ($357, $610)	**$988** ($783, $1,226)	**$71** ($56, $88)
**Total**	**$724** ($540, $982)	**$2661** ($2,412, $2,936)	**$3384** ($3,047, $3,752)	

The 95% confidence intervals were generated from the probabilistic sensitivity analysis.

### Cost-effectiveness

The average per participant implementation costs were compared to the average per participant improvement in HbA1c associated with the intervention to generate the incremental cost per one-point improvement in HbA1c (Table 3). On average, the intervention equated to 71 USD in implementation costs per participant and a 0.26 improvement in HbA1c. Dividing the per participant implementation costs by the per participant improvement in HbA1c generated an incremental cost-effectiveness ratio for *Lifestyle Africa* of 271 USD per one-point improvement in HbA1c. Cost-effectiveness estimates are presented per participant (rather than per club) because the change in HbA1c was assessed at the participant level. As evidenced by the results from the one-way sensitivity analysis (Table 4), the cost-effectiveness was primarily driven by the effectiveness of the intervention on reducing HbA1c.

**Table 3.** Cost-effectiveness of *Lifestyle Africa* (USD).

	Implementation costs	HbA1c
*Lifestyle Africa*	$71* ($56, $88)	6.23* (6.12, 6.34)
Control	0	6.49* (6.37, 6.58)
Incremental (*Lifestyle Africa* – control)	$71* ($56, $88)	−0.26* (−0.11, −0.40)
Incremental cost per point reduction in HbA1c	**$271** **($161, $698)**	

Implementation costs do not include start-up costs. Values in parentheses represent 95% confidence intervals around each average. The intervals were generated from the probabilistic sensitivity analysis.

*Per participant.

**Table 4.** One-way sensitivity analysis results.

Input	Lower input	Lower input ICER	Upper input	Upper input ICER
Intervention average HbA1c	6.12	$191	6.34	$468
Community health workers per club	1	$247	2	$294
Participants per club	5	$257	27	$291
Average hours per session	3.66	$258	5.42	$285
Community health worker hourly wage	$1.63	$258	$2.42	$285
SMS messages per patient	319	$263	704	$280
Administrative coordinator hours per month	4.88	$265	$7.23	$277
Administrative coordinator hourly wage	$2.28	$265	$3.37	$277
Manager hours per month	1.6	$266	2.4	$276
Manager hourly wage	$5.53	$266	$8.19	$276

ICER = incremental cost-effectiveness ratio.

**Figure 1.**
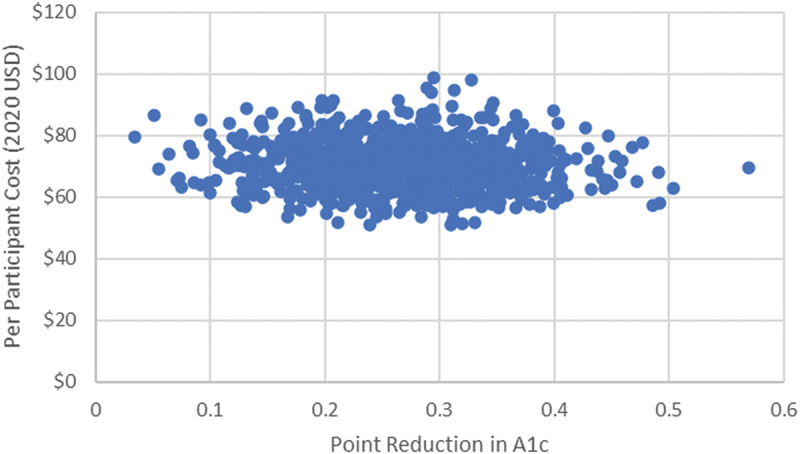
Per participant cost per point reduction in HbA1c: results from the probabilistic sensitivity analysis.

Figure 1 plots each iteration of the probabilistic sensitivity analysis to showcase the potential cloud of cost-effectiveness. The incremental implementation costs ranged between $56 and $88 per participant, whereas the incremental per participant improvements in HbA1c ranged between 0.1 and 0.4. Relatedly, the incremental cost per point reduction in HbA1c ranged from $161 to $698.

## Discussion

The *Lifestyle Africa* programme is associated with improvements in HbA1c and was associated with relatively low costs to implement. Studies in both high-income and low- and middle-income countries have documented the high cost of diabetes care to health systems [21–23] as well as significant cost savings from reducing HbA1c [24,25]. For example, a study that utilised a large US claims database indicated that a one-point reduction in HbA1c among patients with T2DM resulted in a 13% reduction in diabetes care costs [25]. A study of South African healthcare costs estimated an average *annual* cost of care for diabetes management of approximately $825 per patient [22]. If a 13% reduction in diabetes care costs for a one-point improvement in HbA1c was also consistent for the South African population, the implementation cost of *Lifestyle Africa* would be recouped in direct diabetes care cost savings alone within a few years. This benefit depends however on whether reductions in HbA1c can be sustained at no or low cost. There is evidence that lifestyle intervention effects can be sustained for years, but evidence of diminishing effects over time also suggest that maintenance strategies are warranted [26]. Additional research is needed to demonstrate the long-term benefits of the programme.

One of the distinguishing features of the *Lifestyle Africa* programme is that it was designed to be delivered by lay individuals (CHWs) which is more feasible for LMICs. Although few other studies have reported costs of similar lifestyle intervention programmes in LMICs utilising lay providers, the Kerala Diabetes Prevention programme in India that utilised peer leaders found the programme was associated with an intervention cost of about $20 (in 2018 USD) per participant [10]. A study in an under-resourced community in South Africa of a four-session group diabetes education programme delivered by highly trained community member employees to individuals with T2DM that had a significant effect on blood pressure, similarly cost $22 per participant in USD [9].

The *Lifestyle Africa* intervention is somewhat more expensive than these two programmes which appears to be due in part to greater costs associated with programme management/supervision and the cost of delivery of an extensive text-message system. The SMS system selected for programme implementation within this trial was one of the most expensive options, a US commercial product with rich features for scheduling and user permissions as well as global access by all team members. A cheaper option, such as utilising WhatsApp on a local device with a third-party WhatsApp scheduler application that costs less than $5.00, could be used instead. This would dramatically reduce the start-up costs required. Because the *Lifestyle Africa* programme was delivered as a single package, the extent to which the text messaging system improved outcomes and is worth the additional cost is also unclear and should be examined in future research. Programme management or organisational costs were necessary in this study because we partnered with two NGOs to adopt the programme which was not the case for these comparator studies. It is likely that these costs could be reduced as the programme is established within organisations and less management and supervision is needed.

Regardless of the difference in costs between these studies, collectively they suggest that lifestyle interventions delivered by non-professional interventionists may hold promise to improve diabetes management and potentially reduce diabetes-related healthcare costs. This is notable because these early trials in LMICs including the *Lifestyle Africa* trial failed to show significant effects on their primary outcome of weight loss. Nevertheless, because of the low cost of these interventions, the significant effects on secondary outcomes such as HbA1c, blood pressure and other risk indicators show promise of being valuable interventions.

The limitations of this study include the retrospective nature of the cost data, the lack of evidence to support cost-effectiveness related to weight loss and the lack of assessment of changes in other healthcare costs such as medication use. There are also limitations to the potential generalisability of these findings. Our study included two NGOs, which on average provided the programme content to 17 clubs and on average included 14 participants per club. We identified implementation costs at the club level and present estimates based on the average number of participants per club, despite not all of the implementation costs being variable costs. As such, these costs should be interpreted as average costs, and we have provided a plausible range for these costs to capture the potential for variability. It is possible that the resources expended in this study may differ if the intervention was implemented in a real-world setting; however, we studied two NGOs that used different practices to capture heterogeneity. Further, we conducted a probabilistic sensitivity analysis and varied the number of units and unit costs over plausible ranges to attempt to capture this uncertainty. Further, our analysis was conducted in South Africa. The unit costs for equipment and the hourly wages for personnel may differ in other geographic settings. These include potential economies of scale with start-up costs as the number of initial investments (e.g. SMS system) could be re-used multiple times. The findings should also be generalised cautiously to demographic groups other than older women.

Our analysis also does not consider the cost of adapting the programme to other low- and middle-income settings. The materials are freely available at lifestyleafrica.info so the main costs would involve translating the session scripts into the local language, dubbing or filming a presenter reading the narration parts of the script, and adjusting the handouts to reflect any regional food preferences. If desired the still photography and/or animation scenes of the community could be replaced with local scenes and foods. Costs would vary significantly depending on the extent of the adaptation and the local costs of these services. The impact of the cost of adaptation would depend on the scale with which the programme was implemented. If used over time at a large scale, such as a region or country, these would likely have a negligible effect on cost-effectiveness.

## Conclusions

In spite of these limitations, the results indicate the *Lifestyle Africa* intervention has the potential to improve participants HbA1c at a modest cost. More work is needed to strengthen the effectiveness of lifestyle interventions in LMICs given their potential to help address the rising burden of chronic disease in a cost-effective approach.

## Data Availability

The data supporting the conclusions of this article have been made publicly available within Table 1 of this manuscript.

## Paper context

Little is known about cost-effectiveness for delivering community-based lifestyle interventions in low- and middle-income countries. The *Lifestyle Africa* intervention equated to 71 United States dollars (USD) in implementation costs per participant and a 0.26 improvement in haemoglobin A1c (HbA1c) per participant. Decision makers should consider the comparative clinical effectiveness and cost-effectiveness of Lifestyle Africa when making resource allocation decisions.

## References

[1] Kruger HS, Venter CS, Vorster HH. Physical inactivity as a risk factor for cardiovascular disease in communities undergoing rural to urban transition: the THUSA study. Cardiovasc J S Afr. 2003;14:16–9. Epub 2003/03/07. PubMed PMID: 12621539. 12621539

[2] Popkin BM. The nutrition transition and obesity in the developing world. J Nutr. 2001;131:871S–873S. 11238777 10.1093/jn/131.3.871S

[3] Bommer C, Sagalova V, Heesemann E, Manne-Goehler J, Atun R, Bärnighausen T, et al. Global economic burden of diabetes in adults: projections from 2015 to 2030. Diabetes Care. 2018;41:963–970. 29475843 10.2337/dc17-1962

[4] Chen S, Kuhn M, Prettner K, Bloom DE. The macroeconomic burden of noncommunicable diseases in the United States: estimates and projections. PLoS One. 2018;13:e0206702. 30383802 10.1371/journal.pone.0206702 PMC6211719

[5] Ogurtsova K, Da Rocha Fernandes JD, Huang Y, Linnenkamp U, Guariguata L, Cho NH, et al. IDF diabetes atlas: global estimates for the prevalence of diabetes for 2015 and 2040. Diabetes Res Clin Pr. 2017;128:40–50. 10.1016/j.diabres.2017.03.024 28437734

[6] Pheiffer C, Pillay-Van Wyk V, Joubert JD, Levitt N, Nglazi MD, Bradshaw D. The prevalence of type 2 diabetes in South Africa: a systematic review protocol. BMJ Open. 2018;8:e021029. 10.1136/bmjopen-2017-021029 PMC6089280 29997140

[7] Schutte AE. Urgency for South Africa to prioritise cardiovascular disease management. Lancet Glob Health. 2019;7:e177–8. 30528532 10.1016/S2214-109X(18)30476-5

[8] Diabetes Prevention Program Research Group. Reduction in the incidence of type 2 diabetes with lifestyle intervention or metformin. New Engl J Med. 2002;346:393–403. 11832527 10.1056/NEJMoa012512 PMC1370926

[9] Mash R, Kroukamp R, Gaziano T, Levitt N. Cost-effectiveness of a diabetes group education program delivered by health promoters with a guiding style in underserved communities in Cape Town, South Africa. Patient Educ Couns. 2015;98:622–626. 25641665 10.1016/j.pec.2015.01.005

[10] Sathish T, Oldenburg B, Thankappan KR, Absetz P, Shaw JE, Tapp RJ, et al. Cost-effectiveness of a lifestyle intervention in high-risk individuals for diabetes in a low- and middle-income setting: trial-based analysis of the Kerala Diabetes prevention program. BMC Med. 2020;18. DOI:10.1186/s12916-020-01704-9. PMC7472582 32883279

[11] Catley D, Puoane T, Tsolekile L, Resnicow K, Fleming KK, Hurley EA, et al. Evaluation of an adapted version of the diabetes prevention program for low- and middle-income countries: a cluster randomized trial to evaluate “Lifestyle Africa” in South Africa. PLoS Med. 2022;19:e1003964. 35427357 10.1371/journal.pmed.1003964 PMC9053793

[12] Muka T, Imo D, Jaspers L, Colpani V, Chaker L, van der Lee SJ, et al. The global impact of non-communicable diseases on healthcare spending and national income: a systematic review. Eur J Epidemiol. 2015;30:251–277. 25595318 10.1007/s10654-014-9984-2

[13] Roth GA, Mensah GA, Johnson CO, Addolorato G, Ammirati E, Baddour LM, et al. Global burden of cardiovascular diseases and risk factors, 1990–2019: update from the GBD 2019 study. J Am Coll Cardiol. 2020;76:2982–3021. 33309175 10.1016/j.jacc.2020.11.010 PMC7755038

[14] Catley D, Puoane T, Goggin K, Tsolekile LP, Resnicow K, Fleming K, et al. Adapting the diabetes prevention program for low-and middle-income countries: preliminary implementation findings from lifestyle Africa. Transl Behav Med. 2020;10:46–54. 31909412 10.1093/tbm/ibz187 PMC7020390

[15] Catley D, Puoane T, Tsolekile L, Resnicow K, Fleming K, Hurley EA, et al. Adapting the diabetes prevention program for low and middle-income countries: protocol for a cluster randomised trial to evaluate ‘Lifestyle Africa’. BMJ Open. 2019;9:e031400. 10.1136/bmjopen-2019-031400 PMC6858109 31719084

[16] Malhotra R, Hoyo C, Østbye T, Hughes G, Schwartz D, Tsolekile L, et al. Determinants of obesity in an urban township of South Africa. S Afr J Clin Nutr. 2008;21:315–320.

[17] Statistics South Africa. Census 2011 statistical release. Pretoria, South Africa; 2012.

[18] Chapel JM, Wang G. Understanding cost data collection tools to improve economic evaluations of health interventions. Stroke Vasc Neurol. 2019;4:214–222. 32030205 10.1136/svn-2019-000301 PMC6979867

[19] Husereau D, Drummond M, Augustovski F, de Bekker-Grob E, Briggs AH, Carswell C, et al. Consolidated health economic evaluation reporting standards 2022 (CHEERS 2022) statement: updated reporting guidance for health economic evaluations. BMC Med. 2022;20:23. Epub 20220112. 35022047 10.1186/s12916-021-02204-0 PMC8753858

[20] Hirst JA, Farmer AJ, Ali R, Roberts NW, Stevens RJ. Quantifying the effect of metformin treatment and dose on glycemic control. Diabetes Care. 2012;35:446–454. 22275444 10.2337/dc11-1465 PMC3263873

[21] Bansal M, Shah M, Reilly B, Willman S, Gill M, Kaufman FR. Impact of reducing glycated hemoglobin on healthcare costs among a population with uncontrolled diabetes. Appl Health Econ Health Policy. 2018;16:675–684. 29936685 10.1007/s40258-018-0398-2

[22] Erzse A, Stacey N, Chola L, Tugendhaft A, Freeman M, Hofman K. The direct medical cost of type 2 diabetes mellitus in South Africa: a cost of illness study. Glob Health Action. 2019;12:1636611. 31282315 10.1080/16549716.2019.1636611 PMC7012049

[23] Moucheraud C, Lenz C, Latkovic M, Wirtz VJ. The costs of diabetes treatment in low- and middle-income countries: a systematic review. BMJ Glob Health. 2019;4:e001258. 10.1136/bmjgh-2018-001258 PMC6407562 30899566

[24] Keers JC, Groen H, Sluiter WJ, Bouma J, Links TP. Cost and benefits of a multidisciplinary intensive diabetes education programme. J Eval Clin Pract. 2005;11:293–303. 15869559 10.1111/j.1365-2753.2005.00536.x

[25] Lage MJ, Boye KS. The relationship between HbA1c reduction and healthcare costs among patients with type 2 diabetes: evidence from a U.S. claims database. Curr Med Res Opin. 2020;36:1441–1447. 32643451 10.1080/03007995.2020.1787971

[26] Haw JS, Galaviz KI, Straus AN, Kowalski AJ, Magee MJ, Weber MB, et al. Long-term sustainability of diabetes prevention approaches: a systematic review and meta-analysis of randomized clinical trials. JAMA Intern Med. 2017;177:1808–1817. 29114778 10.1001/jamainternmed.2017.6040 PMC5820728

